# Production of Biodiesel from Underutilized Algae Oil: Prospects and Current Challenges Encountered in Developing Countries

**DOI:** 10.3390/biology11101418

**Published:** 2022-09-28

**Authors:** Adewale Adewuyi

**Affiliations:** Department of Chemical Sciences, Faculty of Natural Sciences, Redeemer’s University, Ede 230, Osun State, Nigeria; adewuyia@run.edu.ng; Tel.: +234-803-582-6679

**Keywords:** algae oil, biodiesel, catalysis, transesterification, underutilized oil

## Abstract

**Simple Summary:**

The production of biofuel, especially biodiesel, from algae oil receives little attention in developing countries due to poor enlightenment on biotechnology, high poverty rates, and poor funding of research. This study focuses on finding a better understanding of the evolving prospects and current challenges facing biodiesel production from algae oil in developing nations. Interestingly, several species of microalgae that can serves as sustainable feedstocks for biodiesel production have been identified in developing nations. It is evident that microalgae oil has physicochemical properties that qualifies it for the production of biodiesel, with fuel properties that meet ASTM and EN standards.

**Abstract:**

Biofuel continues to thrive as an outstanding source of renewable energy for the global community. Several resources have been proposed as sources of feedstocks for biofuel; however, some of these have shortcoming. The use of biomass such as algae as a source of feedstock for biofuel is undoubtedly sustainable and green. Unfortunately, the use of algae oil for biodiesel production is underutilized in developing countries. Therefore, this study focuses on finding a better understanding of the evolving prospects and current challenges facing biodiesel production from algae oil in developing countries. The study revealed that less attention is given to the use of algae oil in biodiesel production due to poor enlightenment on biotechnology, high poverty rates, government policies, business strategies, and poor funding of research. Interestingly, several species of algae that can serve as sustainable feedstocks for biodiesel production have been identified in developing countries. It is evident that algae oil has properties that qualify it for the production of biodiesel with fuel properties that meet both the American Society for Testing and Materials and the European standards for biodiesel.

## 1. Introduction

Biodiesel has long been identified as a sustainable replacement for fossil fuel [1]. It is a biomass sourced fuel with superb properties that make is superior to fossil fuel. As a renewable source of energy, it is clean, sustainable, and environmentally friendly [2]. It is produced from forest and agricultural products, as well as from biomass-based domestic and industrial waste products [3]. Using food and cash crops as feedstock for biodiesel production is more challenging in developing countries battling poverty and other socioeconomic difficulties. Although there have been several approaches suggesting the use bio-industrial wastes, this approach has been limited by a lack of interest from local and multinational industries to develop the approach into a large-scale production in many developing countries, such as those in Africa. Other industrialists and researchers have looked in the direction of agricultural wastes, but the challenges to this solution in developing countries have been poor agricultural infrastructure, inconsistent government support, unfavorable climate change, and substandard personnel. With the new wave of biotechnological development in developing countries, attention is shifting towards the use of algae as an alternative source of lipids required as a feedstock for biodiesel production. It is considered as an alternative to food and cash crops because it is safer, has a faster cultivation rate, and is non-competitive. Presently, algae-sourced lipids are the major feedstock used in biodiesel production [4], which has caused Brazil to become the largest producer of biodiesel in the world [4].

Algae is a photosynthetic eukaryotic organism that is non-flowering and typically aquatic [5]. It ranges from unicellular to multicellular forms, growing up to 50 m in length. It has the capacity to germinate without much concern for waste nutrients [6]. Algae cultivation strategies are exploited as a means for direct energy sourcing [7]. In comparison, the lipid yield from an algae cell is higher than that of palm kernel and soyabean cells [8,9]. The oil content varies from about 20 to 80%, which substantiates the use of algae oil as a substantial feedstock for biodiesel production. A study has shown that microalgae have a lipid yield in the range of 30 to 40% [4], which further supports its use as a source for the feedstock of biodiesel production. They can be cultivated in large scale as lipid source for biodiesel.

Biodiesel from algae oil can be classified as a third-generation biofuel. Algae yield more energy per acre of land cultivated than plant crops cultivated per equal acre of land. This has promoted interest in algaculture for the production of biodiesel. Algae oil can be a suitable starting material for biodiesel production [10]. However, there are certain challenges hampering the successful use of algae oil for the production of biodiesel in developing countries. Therefore, this study aimed at understanding the prospect and challenges faced in developing countries using algae oil for the production of biodiesel. This study was conducted by reviewing published peer-reviewed research articles, conference proceedings, short communications, and patents from 1995 to 2021.

## 2. Concept of Algae Oil as a Source of Biodiesel

Biofuel from algae, especially microalgae, is referred to as a third-generation biofuel, which plays a vital role in sustainable energy development. Algae are found in saline and freshwater environments, as well as in sewage systems. Algae require about 2 to 6 days for a complete growth cycle. A short growth period, coupled with their high lipid yield, makes algae—either microalgae or macroalgae—a suitable candidate for a feedstock for biodiesel production. Macroalgae includes brown algae, green seaweed, and red algae. There are over 20,000 kinds of microalgae. When compared with macroalgae, microalgae possess a simpler structure, faster growth, and higher lipid yield.

Microalgae are photosynthetic microorganisms capable of producing biomass rapidly, with about 50% lipid [11] in the form of triglyceride, and their growth rate is faster than that of terrestrial plants. Triglyceride is the starting material for biodiesel production. Several species of microalgae have been identified for biodiesel production. Some selected species of microalgae for biodiesel production are presented in Table 1. They are unicellular of about 1 and 50 µm in diameter, having several classifications, ranging from about 200,000 to 800,000 species [12].

**Figure 1:**
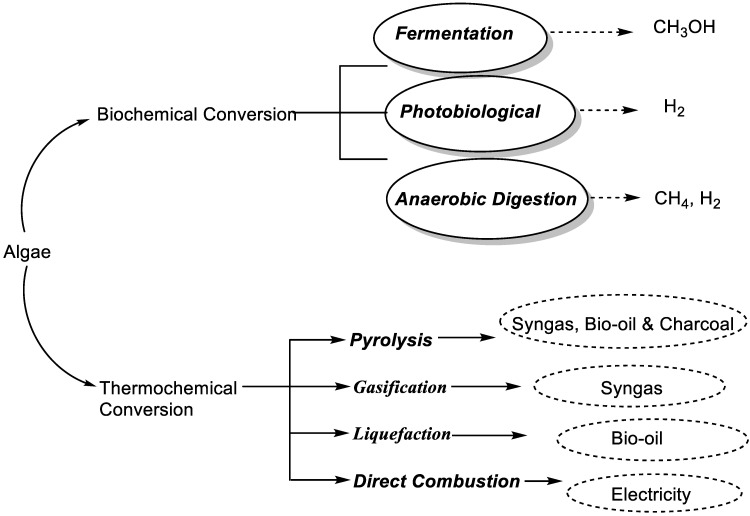
Algae biomass conversion process.

Cultivating and harvesting microalgae are crucial stages in achieving its use as a biodiesel source. A previous study has shown that the harvesting of algae species may account for 20 to 30% of the production cost [13]. The triglyceride content can be converted to biodiesel via the transesterification reaction [14]; however, the conversion of microalgae whole biomass to biofuel can be classified as thermochemical [15,16] and biochemical [17,18] processes, as described in Figure 1. Apparently, several other forms of biofuels can be obtained from algae, which makes it a multisource for different forms of biofuel.

Two main types of microalgae are known—the filamentous and the phytoplankton. In terms of relative abundance, three prominent families of microalgae have been identified: Chlorophyceae (green algae), Bacillariophyceae (diatoms), and Chrysophyceae (golden algae). The cultivation of microalgae has taken different turns over the years, but the most common technique are the use of open ponds and photobioreactors [19]. Microalgae are cultured in the temperature range of 17 to 22 °C. The open pond technique is less expensive and is the most practiced method in developing countries; however, it is very vulnerable to contamination. Several methods have been used for harvesting microalgae, including flocculation, flotation, gravity sedimentation, filtration, electrophoresis, and filtration. These methods are sometimes used in combination to achieve optimum results. Furthermore, the method(s) selected for its harvesting depends on the properties of the microalgae. Oil extraction from microalgae is an important step in preparing the feedstock for biodiesel production [20]. The extraction can be carried out in two steps: mechanical crushing and solvent extraction. Other methods previously proposed for algae oil extraction include pyrolysis, sonication, autoclaving, and microwaving. However, solvent extraction and supercritical CO_2_ fluid extraction remain the most commonly used methods of extraction. The oil yield of some selected microalgae is presented in Tabe 1. The yield varies for different species, suggesting that microalgae is an excellent lipid source for biodiesel production. The various reported fatty acid compositions for different microalgae oils are shown in Table 2. Common fatty acids from microalgae oil include oleic, palmitic, linoleic, stearic, and linolenic acids. The oils from microalgae are predominantly polyunsaturated, which are prone to oxidation. The susceptibility of the oil to oxidation or oxidative rancidity is a concern when considering the shelf-life or stability of biodiesel produced from microalgae oil. Based on quality composition and oil yield, microalgae such as *Chlorella vulgaris*, *Chlorella protothecoides*, *Nannochloropsis* sp., *Nitzchia* sp., *Chlamydomonas reinhardtii*, *Schizochytrium* sp., *Scenedesmus obliques* and *Neochloris oleabundans* have been identified as good sources for biodiesel production [21].

A study on *Symbiodinium clade* C revealed an oil yield of 38.39 ± 6.58% [23]. Oil was extracted from Anabaena PCC 7120 via the solvothermal microwave technique, which revealed a yield of 10.14% per gram of dry biomass [24]. The oil obtained from Anabaena PCC 7120 was subjected to biodiesel production using a titanium oxide catalyst, providing a biodiesel yield of 98.41%. Another study reported an oil yield of 42% from *Neochloris oleoabundans*, with major fatty acids composition being oleic, palmitic, and linoleic acids [25]. The oil was converted to biodiesel via an ultrasonic-assisted transesterification method, with a biodiesel yield of 91%. *Spirulina* sp. oil has also been reported to produce biodiesel containing oleic, palmitic, linoleic, and stearic acids as the major fatty acid contents [26]. Biodiesel has been prepared from microalgae *Botryococcus*, with a biodiesel yield of 84% [27,28,29]; analysis of the biodiesel from *Botryococcus* using gas chromatography and nuclear magnetic resonance revealed the presence of palmitic, oleic, elaidic, and stearic acids. A study on *Chlorella protothecoides* showed an oil yield of 55% in a process that combined bioengineering and transesterification for biodiesel production [30]. Biodiesel produced from microalgae oil has exhibited properties similar to those of petro-diesel fuels and are in line with the standards recommended by the American Society for Testing and Materials (ASTM) and the European standard for biodiesel (EN).

## 3. Challenges and Prospects

### 3.1. Microalgae Processing for Biodiesel Production

The common methods used for the cultivation of algae include open ponds, closed photobioreactors, and hybrid systems. Photobioreactors are the most commonly used methods in developed countries. Based on structural design, photobioreactors may include airlift, tubular, stirred tank, torus, conical and column photobioreactors [29,31,32]. The tubular forms are the most commonly used photobioreactors in developed countries, since they are most suitable for the outdoor microalgae cultivation. They have a large surface area for biomass production. They include inexpensive with simple production designs for the optimum utilization of sunlight. Although they are affordable, local algae cultivators in very low-income countries in Africa still find it difficult to make use of standard tubular photobioreactors due to their inability to afford the overall operational cost; therefore, they improvise with locally sourced materials, which makes the process prone to contamination of all kinds. This has been a challenge to productivity and yield, making it difficult and discouraging for local engagement in algae agriculture in developing countries.

The cultivation of microalgae in open ponds may include the use of natural and artificial pond systems. One major advantage of this method in developing countries is the simplicity and low-cost of production. The production yield is amazingly high, as long as contamination from other sources is minimized. Although waste from sewage and water treatment plants can serve as sources of nutrient supply for the algae ponds, unfortunately, most developing countries still lack adequate structures for handling wastes from sewage and water treatment plants, creating a disadvantage for developing countries with poorly structured waste management schemes. The method is further prone to the effects of climate change. With the current devastating global impact of climate change, the practice of open ponds in developing countries is at a disadvantage, facing severe challenges. For example, there is currently a drought in the Eastern African countries (Ethiopia, Somalia, and Kenya), while some countries in Western Africa (Nigeria, Ghana, and Gambia) presently suffer from flooding. Environmental pollution from gases released from industrial waste has contributed to the challenges faced by algae cultivation using the open ponds. Examples include countries in Asia, due to rapid industrial growth. Countries such as China and India are at the receiving end, where the pH and salinity of environmental water sources change due to the level of dissolved gases from the environment. The abrupt change in pH and water chemistry affects the practice of using open ponds for algae cultivation in such countries. Unfortunately, most local algae farmers cannot afford the more advanced algae cultivation processes. There may be a need to pretreat open water resources used for biofuel cultivation. The pretreatment varies, depending on the nature of the water source and the level of contaminants compared to microalgae growth in the water. The pretreatment given to the water resource is an additional measure that increases production costs.

**Figure 2:**
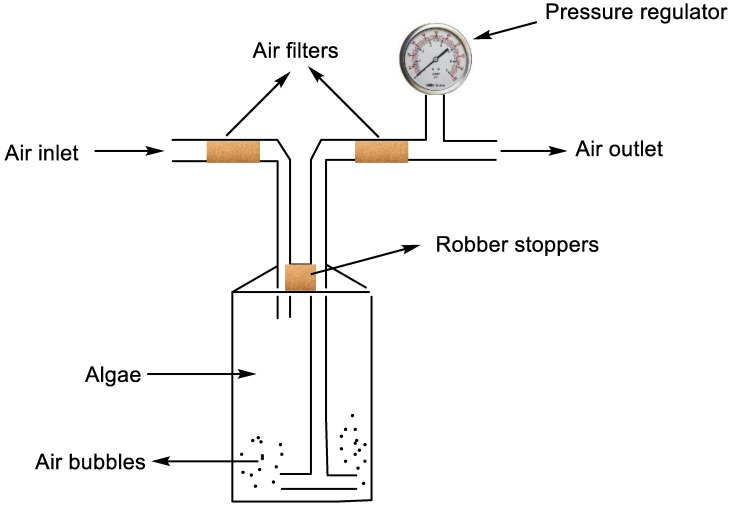
Laboratory-scale photobioreactor model for cultivating algae.

Currently, research is ongoing for the development of procedures that will be cheap and appropriate for the cultivation of algae with a high lipid yield. Figure 2 shows a simple laboratory-scale photobioreactor model for cultivating algae. Although several other closed-door methods are used alone, or to complement the open pond method [33], they are not still as cheap as using the natural open pond method. Developing or simplifying the currently used approaches to make them affordable in low-income countries is still challenging for researchers. This might be a good area of research worth investigating for scientists in developing countries.

**Figure 3:**
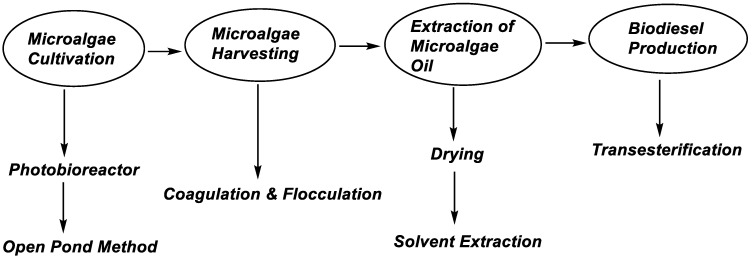
Description of biodiesel production from microalgae in developing countries.

Apart from cultivation, the processes employed in developing countries for harvesting microalgae also face some challenges, which include their design, management, and cost. Some popular harvesting methods are electrophoresis, ultrafiltration, coagulation, centrifugation, filtration, flocculation, and air-flotation [34,35]. Other methods are currently being developed or modified from time to time. The method chosen is usually based on the cultivation process and the desired product. For biodiesel production in most developing countries, coagulation and flocculation are commonly used. One major challenge faced when using these approaches in developing countries is the process of energy consumption. The coagulation process is mainly an ‘electrolytic coagulation process,’ which requires energy intake. The provision of electrical energy is always disrupted in low-income countries. Therefore, the provision and supply of a stable source of electricity come at an extra price, increasing process costs. However, it is important to minimize energy consumption as much as possible. Sometimes, the combination of coagulation and flocculation is used. The combined methods help reduce the number of chemicals used in the harvesting process and the process time for effective results. The microalgae obtained can then be dried before proceeding with oil extraction using suitable solvents and transesterification for biodiesel production. The steps involved in most developing countries are described in Figure 3; these steps may be different for more advanced processes in developed countries.

The harvesting process may be a batch process, continuous, or semi-continuous process, depending on production scale and cost [36,37]. The batch process is easy to control; however, the cost evaluation of the continuous or semi-continuous process is cheaper for long-term usage. Despite the fact that the continuous and semi-continuous processes are cheaper, sustaining them in developing countries is difficult due to poor technology and infrastructure development. Table 3 compares batch and semi-continuous processes for some selected microalgae. The table shows the amount of microalgae produced per culture medium (amount of biomass, g L^−1^), oil yield from the microalgae obtained (% wt wt^−1^), and the amount of oil obtained from the cultivated microalgae medium per day (oil production, mg L^−1^ day^−1^). Obviously, the final oil production from the semi-continuous process is higher than that obtained using a batch process. There may be the need to pay more attention to developing simple and affordable semi-continuous processes for developing countries to promote the use of microalgae oil, especially for its use as a feedstock for biodiesel production.

### 3.2. Availability of Microalgae Species

Microalgae biotechnology is receiving significant attention in some low-income countries due to recent prospective applications in pharmaceutical, bioremediation, food, and nanotechnology fields [42]. Many developing countries have reported several species of high oil-yielding microalgae [43]. Most developing countries have climatic conditions that favor the survival of the microalgae. Moreover, the weather conditions and level of natural light penetration, CO_2_ availability, water, and temperature in most developing countries in Africa and Asia favor natural habitats for microalgae survival. These are encouraging factors that promote the use of microalgae in developing countries. In a theoretical estimation, microalgae can utilize 9% of incoming solar irradiation to generate 280 tons of dry biomass per hectare per year, while using 513 tons of CO_2_ [42,44]. With the abundance of sunlight in developing countries, the survival of microalgae is certain. Apart from this, microalgae can help reduce the fear of the greenhouse effect due to pollution from CO_2_. Microalgae thrive in wastewater, saline, and brackish water environments. A large amount of wastewater generated can find application in microalgae cultivation in developing countries. Furthermore, microalgae can survive or be easily cultured in saline or brackish water systems in low-income countries in the Middle East, where there is limited supply of natural freshwater. Among the microalgae found in developing countries are *Chlorella pyrenoidosa,* *Prymnesium parvum, Tetraselmis chuii, Tetraselmis suecica, Isochrysis galbana, Tetraselmis suecica*, *Chlorella stigmatophora, Nanochloropsis gaditana, Nanochloropsis oculate, Euglena gracilis,* *Botryococcus braunii*, *Neochloris oleoabundans, Phaeodactylum tricornutum,* and *Dunaliella tertiolecta*. However, freshwater-sourced microalgae, such as *Oleoabundans* sp., and marine microalgae, such as *Nannochloropsis* sp., are well known as good sources of oil for biodiesel production.

Currently, Egypt is the leader in aquaculture in Africa, with about 987 tons produced in 2011, and even higher production estimating for the coming years [42,45,46]. Microalgae are known to serve as a food source for the larvae of fish and crustaceans. However, in the past, little attention has been paid to microalgae as a possible source of oil for biofuel. Interestingly, with the abundance of different microalgae species in developing countries, the future is bright for biofuel production from a renewable resource with the potential of being sustainable in the developing world. The currently envisaged challenge might be a competitive demand for microalgae as an animal feed and colorant for crustaceans [42,47,48]. As of 2004, the market for microalgae-sourced colorant was valued at USD 200 million, with an estimated USD 2500 per kilogram [47]. Pigments such as phycobilins have been isolated in microalgae [49]. A study reported the biosynthesis of phycocyanin (blue pigment) by *Spirulina platensis* [50]. In Asia, China is one of the countries where microalgae, such as cyanobacteria Nostoc, are used as food. A similar case was found in the Republic of Chad, where cyanobacteria Arthrospira are considered to be a food source [42]. The nutritional value of *Nostoc sphaeroides* as food has been reported [51]. Furthermore, *Chlorella*, *Spirulina*, *Tetraselmis*, *Isochrysis,* and *Nannochloropsis* have been reported for their nutritional (human consumption) and bioactive potentials [52]. These microalgae have exhibited antioxidant, antihypertensive, antidiabetic, antihyperlipidemic, and immunomodulatory capacities in human and other animals [52]. This multi-functionality of microalgae has made them competitive as foods and colorants, which is an emerging limitation challenging their global use as a feedstock for biodiesel production. This is not only a challenge in developing countries, but is also a global phenomenon.

### 3.3. Government Policy and Business Strategy

Government policies on biofuel are at different developmental stages in different countries of the world. Governments in countries with a large deposit of fossil fuels are reluctant to enact policies that favor biofuel production due to their dependence on crude oil fossil for income revenue. The lack of willpower to support policies promoting biodiesel production growth is a significant challenge in most developing countries. Despite the global progress made in biogenetic engineering to drive gene modification, there is still an underrepresentation of skill acquisition in this field in developing countries. There is limited state-of-the-art equipment to drive research or large-scale modification of microalgae genes for industrial production. It cannot be emphasized enough that genomics can play an important role in boosting the profile of microalgae for biofuel production. A recent study reported the genetic modification of microalgae as a means of enhancing biorefinery [53]. Various studies have reported different approaches to genetic modification to improve lipid biosynthesis in microalgae, as shown in Table 4. Unfortunately, most of these reports from research endeavors remain merely as published articles or chapters in books, without being used in real-life applications. It is important that governments in developing countries help bring research findings on biofuel into commercial application by creating enabling environment in terms of providing suitable legal frameworks and financial support.

On the other hand, poor business strategy is also hampering the growth of biofuel from microalgae in developing countries. The government’s approach towards developing local energy industries is flawed. The government needs to ease the tax rate on biofuel. In Nigeria, the government pays subsidies on petroleum from fossil fuels, whereas this is not the case for biofuel. Presently, the petroleum pump price in Nigeria is about 50% subsidized by the government. If similar support is given to biofuel sales, it will go a long way towards promoting biofuel and a green environment. Most developing countries are not attractive to foreign investors, due to a lack of security. A good example is the case of social unrest and ethnic clashes in Africa. Economic instability relating to social insecurity has discouraged several multinational companies from investing in the biofuel business in some developing countries. This challenge has a negative impact on biofuel commercialization in developing countries. Another factor is the fluctuation in local currency. Local investors risk losing financial strength due to the conversion rate of local currency to the dollar, which is the international trading currency. Many local industries in developing countries have folded up due to a sudden crash in the bargaining power of local currency against the dollar. It is important to create a business-enabling environment for biofuel by putting a legal framework in place that will help formulate policies that promote biofuel’s survival in developing countries.

### 3.4. Economic Feasibility and Commercialization

The strength, weakness, opportunity, and threat (SWOT) analysis, and life cycle assessment to understand the advantages and disadvantages of sourcing biofuel from microalgae have been reported [63]. It became apparent that the production of biofuel from microalgae will consume a significant amount of energy. It is also certain that microalgae biofuel output is higher than that of other terrestrial crops. However, the production of biofuel from microalgae requires more energy consumption than other biomass feedstock. It is also known that some green gases may be released into the environment during the processing of microalgae for biofuel. Therefore, such gases must be contained in order to minimize pollution and process contamination. Another study revealed that the cultivation of microalgae may require the use of fertilizer, which is an additional cost [64]; when such fertilizer is nitrogen or phosphorus-based, any excess nitrogen or phosphorus must be recycled. The amount of water required for the cultivation is more than what is required for the cultivation of other biomass feedstocks; a previous publication from the National Academy of Sciences of the United States of America revealed that about 3.15 to 3.65 liters of water are required to produce 1 liter of microalgae biofuel [63]. Furthermore, about 39 billion liters of algae oil can be generated using 123 billion liters of water. Therefore, the suggestion that a high amount of water and fertilizer supply is required for microalgae cultivation for biofuel production is critical [65]. The lack of sustainable water resources has been a challenge in low-income countries in the Middle East. However, there has been an effort involve in cloud seeding and water harvesting to boost water availability [66].

The production of biodiesel from microalgae oil is still in its infancy in most developing countries, with the exception of Brazil, China, and Argentina, with more robust prospects for biotechnological advancement in converting microalgae oil to biodiesel. The cost of producing algae biomass in an open pond was previously reported to range between 0.3 to 0.4 € kg^−1^ [67]. However, for the photobioreactor, the cost ranged between 3.8 and 4.5 € kg^−1^ [67]. The significant difference in cost between the open pond and photobioreactor methods was attributed to the higher electricity consumption for the reactor’s mechanical operation. The low production cost must be maintained to effectively commercialize the creation of biodiesel from microalgae oil. When the oil production from the microalgae biomass was considered, it was concluded in a recent study that depending on the method used for expelling the oil, the price ranged from 0.81 to 2.43 USD kg^−1^ [68]. Therefore, many factors, such as pretreatment, electricity consumption, CO_2_ consumption, pressure, etc, will have to be considered before determining on the final cost of production. One significant advantage in developing countries is that the cost of labor per capita is cheap, which helps keep production costs to a minimum. With the high population rate, abundant natural resources for open ponds, and large waste generation per annum, the future of biofuel from microalgae is bright in developing nations.

A previous report revealed that during the pre-Covid-19 period, petroleum diesel was sold for USD 3.24 per gallon, while biodiesel cost USD 3.55 [69,70]. Interestingly, a study conducted an evaluation that compared the cost of producing biodiesel with that of petroleum-based biodiesel and concluded that the production of biodiesel might be estimated as USD 2.29 kg^−1^. In contrast, the production cost of petroleum diesel may be estimated at USD 1.08 kg^−1^ [71]. The higher price of biodiesel from microalgae was attributed to the higher production and operation cost [72]. This showed that the cost of production and processing may be reduced by improving the cultivation and harvesting processes. The profit from microalgae biodiesel is currently low, with an annual benefit of USD 4.82 million generated as revenue [73]. This profit is estimated to increase with the use of wastewater as a water resource for microalgae cultivation [73,74]. More importantly, a study obtained an estimated cost of EUR 2.01 kg^−1^ for the production of microalgae biomass on a 15 hectare of land for small-scale operation, whereas a corresponding estimation of EUR 0.33 L^−1^ was obtained for biodiesel production on the same scale [72,73]. However, an earlier study showed that when biodiesel production was increased from 10,000 to 100,000 tons, the gross production cost was reduced from USD 8.1 to USD 6.3 [73,75]. Therefore, high scale biodiesel production may serve as a means of reducing production costs. With the high population index in most developing countries, the demand for biodiesel from microalgae is expected to be high.

## 4. Current Status and Future Perspective

Several projects have been taken up regarding the use of microalgae to create biofuel in many developed countries; for example, the United States of America green energy program and Arizona Public Service co-established a microalgae production system with a biofuel yield reaching 5000–10,000 gallon per acre per year [63]. As a follow up, the national energy board of the United State launched the ‘Mini-Manhattan Project’ to improve microalgae oil production (Keune, 2012). The European Union has also launched an algae bioenergy development action plan project (EnAlgae), with the aim of improving algae production in Europe [63].

The use of biodiesel as an alternative replacement for petro-diesel is not popular in science and technology lagging countries (STLCs) due to the factors listed above. Major biodiesel producing countries among developing countries are: Indonesia, Brazil, China, Argentina, India, Thailand, South Africa, Malaysia, the Philippines, Ghana, Mexico, Uruguay, Paraguay, Croatia, and Colombia [76,77,78,79,80]. Although fresh and waste cooking oils are the common feedstocks used for biodiesel production in developing countries, the use of microalgae oil is catching up. The development is small in African countries, except for in South Africa, Egypt, Morocco, and Ghana. Economic globallization might be an assured pathway to the solution in developing countries [80]. With the exception of some countries in Asia and South America, laboratory-based research work on the use of microalgae for biodiesel is scarce. This goes mainly for African countries. It is important to note that despite the ongoing awakening going on in developing countries to encourage the use of biodiesel, the process is still hampered by the conditions discussed above.

Among the developing countries, China is playing a significant role in microalgae cultivation projects. In this regard, the Chinese Academy of Sciences has developed efficient techniques for achieving this purpose. Several trainings on scaled-up production have been undertaken [63,81]. Brazil has also made a significant contribution in promoting the creation of biodiesel from microalgae oil [82,83,84]. Currently, the Petrobras company, Brazil, is researching and investing in microalgae production in the petrochemical sector, while the Ministério da Ciência, Tecnologia e Inovação (MCTI), through the Conselho Nacional de Desenvolvimento Científico e Tecnológico (CNPq), is committed to investing significant funding in research endeavors in the tertiary institutions and research institutes to promote cutting edge research on obtaining biofuel from algae [82,84,85]. Countries in Africa are far behind in this regard, and there is a need for them to do more and to become committed to promoting the production and commercialization of biofuel.

Economic instability and lack of technological infrastructure in developing countries are serious drawbacks to the production of biodiesel from microalgae oil. The situation is very poor in developing countries in Europe, such as Moldova, Albani, Bosnia and Herzegovina, Serbia, and Montenegro. Due to poor cultivation of microalgae in most African countries, attention is mainly on the use of waste cooking oil. However, with the current advocacy to promote the eradication of hunger (Sustainable Development Goal 2) by the African Union, attention may shift towards microalgae oil in the coming years in order to prevent overdependence on this food crop, which will go a long way in making the arid land available for growing enough food for the African populace. The rural regions in developing countries are a large reservoir of biological resources, including microalgae [86,87]. The potential of these rural regions is not sufficiently harnessed [87,88].

Most research works published on the use of microalgae oil in biodiesel production are laboratory-based experiments. There is an urgent need to devote more time to the large-scale cultivation of microalgae and its biodiesel production on a commercial scale. There is a need to further investigate the different steps involved in the cultivation and harvesting process in order to help reduce production costs. There is also a need to develop new strategies and techniques for cheap and affordable microalgae processing. Governments in developing countries are expected to be more committed to enacting policies that will drive the course of microalgae oil production and eventually, its biodiesel production. Effort is required to improve government policies that will favor socioeconomic development, motivating foreign investors and multinationals to invest in biofuel businesses in developing countries. It is paramount to provide support in the form of research funding to works that are focused on renewable energy production in order to enhance technological advancement in this area of development.

The level of enlightenment on the prospect of biofuel is poor in developing countries which may be attributed to high poverty rate and poor socioeconomic development. It is necessary to create more awareness regarding the environmental danger associated with the long-term use of fossil fuel energy. There is a need to build on the research capacity of early and young researchers in developing countries. There is dearth of skills in biotechnology and the use of molecular engineering tools. Therefore, there is an urgent need to build greater research capacity in this area. Governments in developing countries need to increasingly partner with developed countries to build relationships that will result in research collaboration, the exchange of skills, and the transfer of technology.

## 5. Conclusions

Microalgae continues to be a promising resource for biofuel generation. This study considers the prospects and current challenges encountered in developing countries regarding the use of microalgae oil as a resource for biodiesel production. Currently, the use of microalgae is receiving significant attention, and there is urgent need to invest in finding a cheaper and more sustainable technique that will promote the cultivation of microalgae for biodiesel production in developing countries. It is important to note that the use of microalgae oil for biodiesel production is at different developmental stages in different developing countries of the world. However, factors such as microalgae processing, poor enlightenment on biotechnology, economic feasibility, government policy, business strategy, high poverty rates, and poor research funding have been identified as factors limiting the production of biodiesel from microalgae oil in developing countries. These factors vary from one developing country to another. The worst scenarios involving all of the factors listed above are found in the least-developed countries (low income countries). Despite the abundant prospects, there is still dearth of technical know-how for achieving the commercialization of biodiesel from microalgae oil in developing countries. There is an urgent need for governments in developing countries to focus on the use of biomass resources such as microalgae as a means to circumvent emerging current and future energy crises.

## Figures and Tables

**Table 1:** Lipid content of some selected species of microalgae.

Algae Species	Oil Content (% Dry Weight)
*Ankistrodesmus TR-87*	28–40
*Botryococcus braunii*	29–75
*Dunaliella tertiolecta*	36–42
*Tetraselmis suecica*	15–32
*Neochloris oleoabundans*	35–54
*Chlorella emersonii*	28–32
*Botyococcus braunii*	25–80
*Chlorella vulgaris*	14–22
*Euglena gracilis*	14–20
*Neochloris oleoabundans*	35–54
*Phaeodactylum tricornutum*	20–30
*Pleurochrysis carterae*	30–50
*Prymnesium parvum*	22–38
*Schizochytrium* *sp*	50–77
*Scenedesmus dimorphus*	16–40

**Table 2:** Major fatty acid composition of some selected species of microalgae [22].

Fatty Acid	*Synechocystis* *pevalekii*	*Spirulina* *plantensis*	*Chlorococcum* *infusionum*	*Cladophora* *crystallina*	*Navicula* *minima*
C14:0	1.00	13.27	8.50	8.80	2.70
C16:0	34.20	21.10	25.62	39.10	26.40
C16:1	3.80	9.32	5.46	2.80	18.70
C18:1	29.80	11.27	15.66	31.80	25.30
C18:2	14.90	0.24	7.50	11.60	2.20

**Table 3:** Comparison of cultivation process for microalgae oil production of some selected species of microalgae reported in literature.

Microalgae	Cultivation Process	Amount of Biomass (g L^−1^)	Oil Yield (% wt wt^−1^)	Oil Production (mg L^−1^ day^−1^)	Reference
*Desmodesmus* sp. F2	Batch	3.32	64.10	263	[38]
*Desmodesmus* sp. F2	Semi-continuous	3.99	45.60	302	[38]
*Chlorella* sp.	Batch	2.15	44.80	124	[39]
*Chlorella* sp.	Semi-continuous	1.10	45.10	139	[39]
*Nannochloropsis oculate*	Semi-continuous	1.00	30.70	151	[40]
*Nannochloropsis* sp.	Batch	3.83	19.30	74	[41]

**Table 4:** Previously reported genetic and metabolic engineering modification of microalgae for the enhancement of lipid biosynthesis.

Microalgal Strains	Genetic Modification	Performance	Reference
*Nannochloropsis oceanica*	Malonyl CoA-acylcarrier protein transacylase.	Neutral lipid content increased by 31%.	[54]
*Chlamydomonas* *reinhardtii*	*C. reinhardtii* transformed with acyl-ACP thioesterases.	Lipid content increased by ~56%.	[55]
*Schizochytrium* sp.	Overexpression of malonyl-CoA: ACP transacylase (MAT) in *Schizochytrium.*	Increase in polyunsaturated fatty acids and lipids by 10.1%.	[56]
*Mychonastes afer*	Cloning and expression of 3-ketoacyl-coA synthase gene from *M. afer* (*MaKCS)in Saccharomyces cerevisiae* BY4741.	Increased lipid content, especially nervonic acid under stress conditions of high light and low nitrogen.	[57]
*Nannochloropsis salina*	Overexpression of basic leucine zipper in *N. salina.*	Improvement in both growth and accumulation of lipid.	[58]
*Nannochloropsis oceanic*	Transposome complex Tn5 containing anti-biotic resistance cassette was inserted in *N. oceania* generating random mutant strain.	High accumulation of intracellular lipids.	[59]
*Chlamydomonas* *reinhardtii*	Phospholipase A2 (PLA2) gene knockout.	Increased lipid production by 64.25%.	[60]
*Chlamydomonas* *reinhardtii*	Overexpression of a DNA-binding-with-one-finger (*Dof*) *transcription factor.*	Increase in fatty acid production in sulfur deficient medium by 15.58% and in nitrogen by 17.02%.	[61]
*Chlamydomonas* *reinhardtii*	Cloning of crDOF from *Chlamydomonas reinhardtii* and construction of transgenic lines. Overexpression of crDOF.	Increased intracellular lipid content.	[62]

## Data Availability

Not applicable.
